# Painless Subacute Thyroiditis in a Patient With Acute COVID-19 Infection: A Transient Event

**DOI:** 10.7759/cureus.26924

**Published:** 2022-07-16

**Authors:** Tsering Dolkar, FNU Jitidhar, Meet J Patel, Abubaker M Hamad, Ferdous Salauddin, Zewge Shiferaw-Deribe, Muhammad H Dogar

**Affiliations:** 1 Internal Medicine, One Brooklyn Health (OBH) Interfaith Medical Center, Brooklyn, USA; 2 Medicine, Memorial Hospital of Converse County, Douglas, USA; 3 Medicine, One Brooklyn Health (OBH) Interfaith Medical Center, Brooklyn, USA; 4 Internal Medicine/Endocrinology, One Brooklyn Health (OBH) Interfaith Medical Center, Brooklyn, USA; 5 Internal Medicine/Cardiology, One Brooklyn Health (OBH) Interfaith Medical Center, Brooklyn, USA

**Keywords:** low tsh, thyroid antibodies, covid-19, tachycardia, subacute painless thyroiditis

## Abstract

Coronavirus 2019 disease (COVID-19) is a highly contagious infectious disease caused by severe acute respiratory coronavirus 2 (SARS-CoV-2). Although several articles have described the non-respiratory effects of COVID-19 in the past two years, there are few reports of COVID-19 associated with thyroiditis. We present a case of a middle-aged female patient with positive COVID-19 PCR associated with acute pulmonary embolism and thyroiditis. Three months ago, her baseline thyroid profile was normal. Thyroiditis induced elevated free thyroxine (FT4) and decreased thyroid-stimulating hormone (TSH) levels resolved with conservative management within six days.

## Introduction

Coronavirus 2019 disease (COVID-19) is a highly contagious infectious disease caused by severe acute respiratory coronavirus 2 (SARS-CoV-2). Although several articles have described the non-respiratory effects of COVID-19 in the past two years, there are few case reports of COVID-19 associated with thyroiditis. We present a case of a middle-aged female patient who presented with generalized abdominal pain and was found to have concomitant painless subacute thyroiditis with acute COVID-19 infection. Three months ago, her baseline thyroid profile was normal. Thyroiditis induced elevated free thyroxine (FT4) and decreased thyroid-stimulating hormone (TSH) levels resolved with conservative management within six days.

## Case presentation

**Figure 1:**
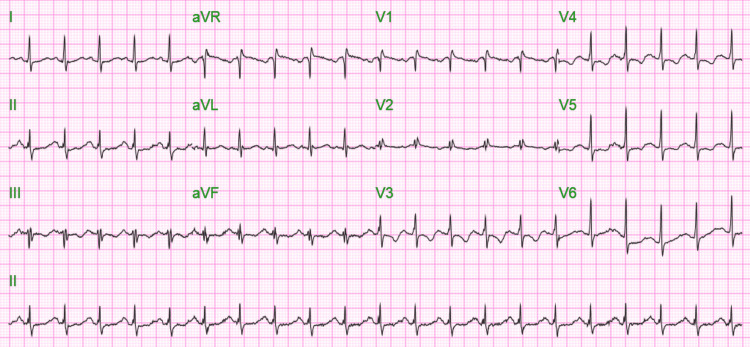
EKG on admission showing sinus tachycardia with a ventricular rate of 125/min and QTc of 574 ms

A 55-year-old African American female with a past medical history of hypertension presented to the emergency department (ED) complaining of generalized abdominal pain for one day. The patient's physical exam was normal including an eye examination. The heart and lung examinations were benign. There was no abdominal tenderness. Triage vitals were blood pressure of 146/106 mmHg, pulse of 126/min regular, temperature of 36.4°C (97.6°F), and oxygen saturation of 86% on room air. The patient was placed on 6 liters/min oxygen support via nasal cannula (NC). On admission, an electrocardiogram (EKG) (Figure 1) showed sinus tachycardia with a ventricular rhythm of 125 beats per minute and prolonged QTc of 574 ms.

**Figure 2:**
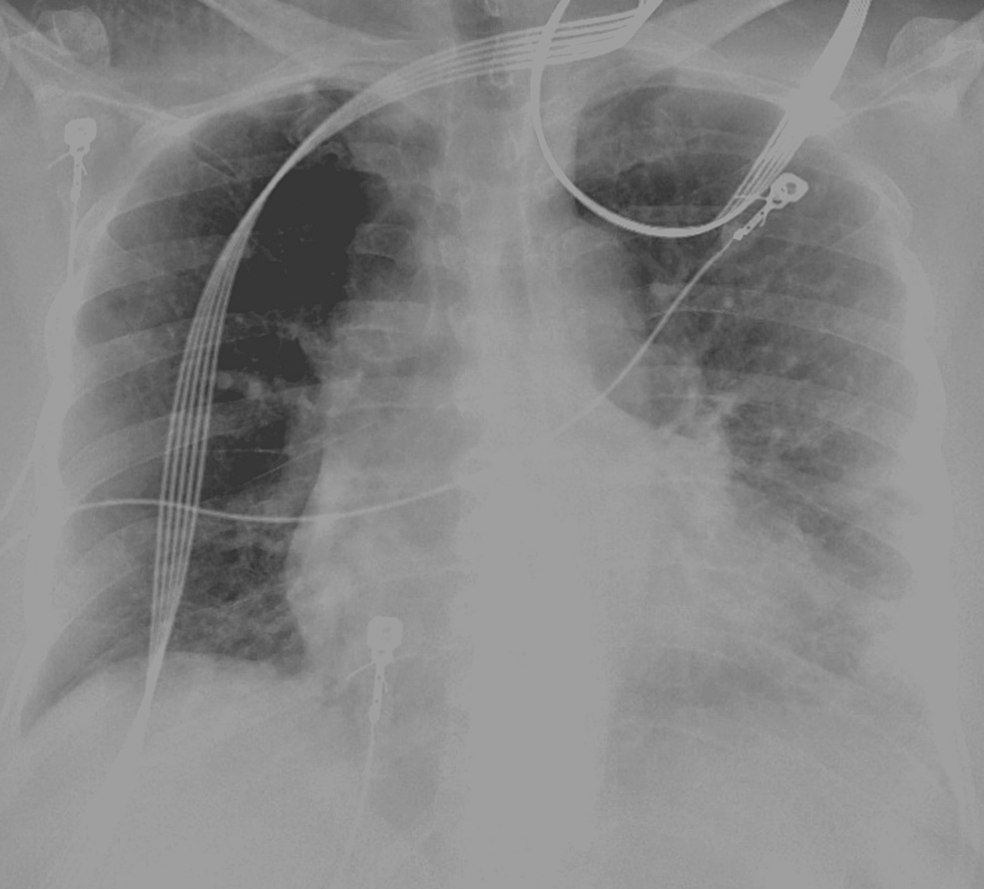
Chest x-ray on admission showing patchy lower left lung infiltrates compatible with pneumonia

A chest x-ray (Figure 2) showed patchy left lower lung infiltrates compatible with pneumonia on admission. She was tested positive for SARS-CoV-2 PCR. D-dimer was elevated to 4727 ng/ml (reference range: 0-500 ng/ml), with high sensitivity troponin of 77.8 ng/L (reference range: 0.0-35.0 ng/L) and brain natriuretic peptide (BNP) of 233.9 pg/ml (reference range: 10.0-100.0 pg/ml). The patient was admitted for acute hypoxic respiratory failure due to COVID-19 pneumonia and suspected pulmonary embolism (PE). Arterial blood gas analysis on 6 liter/minute NC showed normal pH with normal pCO2, normal pO2, and O2 saturation of 94%.

**Figure 3:**
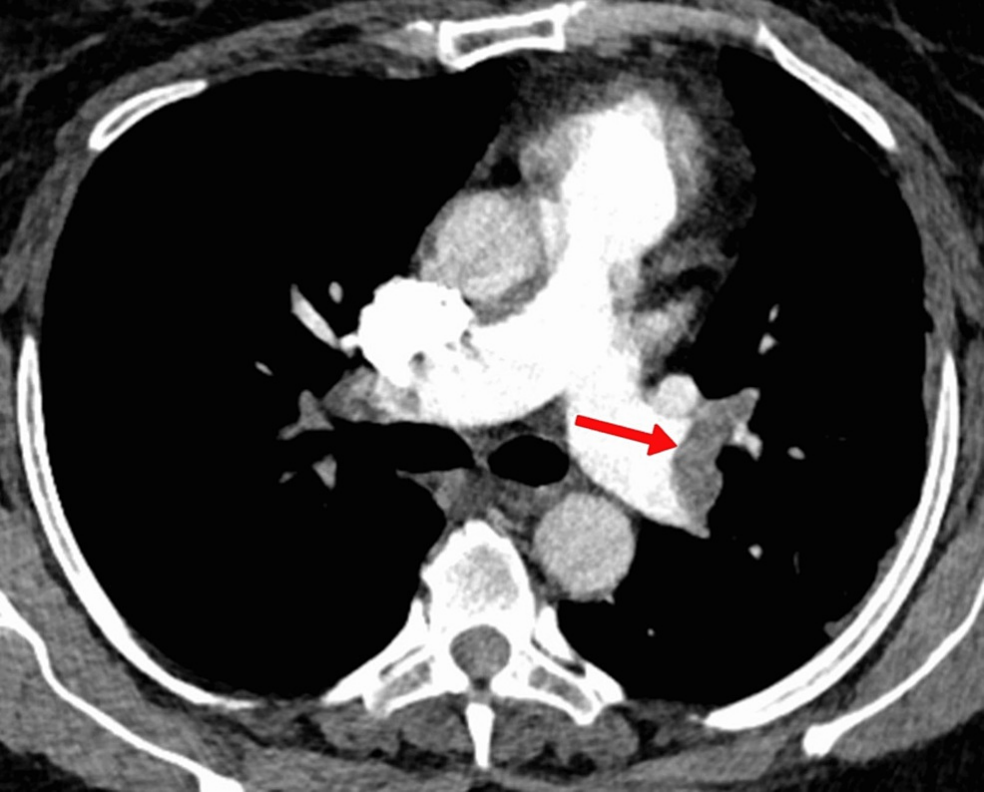
CT angiogram of the chest showing left main pulmonary artery thrombus

CT angiography chest with IV contrast (Figure 3) was done, which showed extensive acute PE extending from the distal right and left main pulmonary arteries into the bilateral upper and lower lobe pulmonary arterial branches and right middle lobe pulmonary arterial branch.

The patient received ceftriaxone 1 gm intravenous once daily, azithromycin 500 mg intravenous once daily, dexamethasone 6 mg intravenous once daily, remdesivir 100 mg intravenous once daily, amlodipine 10 mg per oral daily, and enoxaparin 1 gm/kg body weight subcutaneously for every 12 hours. Abdominal pain likely secondary to gastritis resolved with famotidine.

She had an abnormal thyroid function test, but the patient denied any symptoms of hyper- or hypothyroidism. Her baseline TSH was 1.940 uIU/mL, and her total T4 was 8.7 ug/dL in the test taken at her primary care physician’s clinic three months ago. On day 1, TSH was 0.075 uIU/mL (low), FT4 was 2.51 ng/dL (high), and T3 was 99 ng/dL. Both TSH and FT4 normalized on day 6 (Table 1). Heart rate also trended down during the same time (Table 2). Thyroid antibodies were normal. Thyroid-stimulating immunoglobulin was <0.10 IU/L (normal range: 0.00-0.55 IU/L), thyroglobulin antibody was <1.0 IU/mL (normal range: 0.0-0.9 IU/mL), and thyroid peroxidase antibody (TPO) was 10 IU/mL (normal range: 0-34 IU/mL).

**Table 1:** Trend of the thyroid profile

Day since admission	Thyroid-stimulating hormone (TSH) (Normal: 0.465-4.680 uIU/mL)	Thyroxine (T4) (Normal: 0.78-2.19 ng/dL)	T3 triiodothyronine (Normal: 71-180 ng/dL)
Day 1	0.074	2.51	
Day 4	0.197	1.93	
Day 6	1.030	2.17	99
Day 7	0.617	1.84	94

**Table 2:** Trend of the heart rate during the hospitalization course

Day since admission	Heart rate (beats/minute)
1	126
2	108
3	99
4	97
5	111
6	102
7	117

We treated thyroiditis conservatively. The patient’s oxygen requirement decreased to 2 L NC on day 3; by day 5, she saturated at 98% of room air. We discharged the patient home on oral anticoagulation.

## Discussion

The term thyroiditis means thyroid inflammation. Most endocrinologists classify thyroiditis into (1) autoimmune thyroiditis (Hashimoto's thyroiditis and Graves’ disease); (2) infectious thyroiditis (includes all forms of infection, except viral); (3) Riedel's thyroiditis, and (4) subacute thyroiditis (SAT). Most commonly, subacute thyroiditis is associated with viral infections [1]. SAT commonly presents in either of the two forms: (a) painful SAT or (b) painless SAT. Our patient has atypical or painless subacute thyroiditis associated with COVID-19 infection.

SAT typically presents as pain localized to the anterior aspect of the neck radiating to the jaw or ears, low-grade fever, fatigue, and palpitations. Cardinal signs include tenderness on palpation and enlarged gland size. Laboratory findings suggest hyperthyroid etiology that has suppressed TSH levels, low FT4, and poor or no thyroid uptake. Thyroid ultrasound (USG) shows expected results of poorly defined hypoechoic areas with a heterogeneous echo pattern [2]. There may be a triphasic clinical course of hyperthyroidism and hypothyroidism and a return to normal thyroid function. However, the hypothyroid phase may last for months until the patient becomes euthyroid [3]. Autoimmune and postpartum SAT are painless, where the thyrotoxic phase lasts weeks, not days.

It is believed that SARS-CoV-2 can affect thyroid function in several ways. The three reported effects of the virus are (1) hypothyroidism (central or primary), (2) thyrotoxicosis (either subacute/painful thyroiditis or painless/atypical thyroiditis), and (3) nonthyroidal illness syndrome (previously known as a euthyroid sick syndrome) [4]. This conveys that the effects of the virus on the thyroid gland are highly variable, and it is not easy to predict the abnormalities in thyroid function tests (TFTs). Nonetheless, systematic reviews observe a similar pattern in presentation, TFTs, and outcomes when studying the complication of subacute thyroiditis in COVID-19 [5].

**Figure 4:**
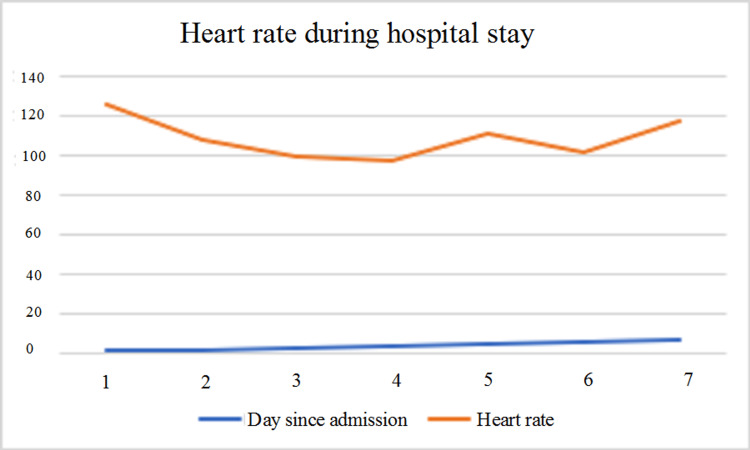
Graphical trend of the heart rate during this admission

Inflammatory markers like erythrocyte sedimentation rate (ESR) and C-reactive protein (CRP) are typically elevated, accompanied by the release of cytokines in thyroiditis [6]. Post-viral immunological reactions have also been described as a cause of thyroid problems [7]. A possible mechanism with evidence is direct viral injury. The molecular interaction of SARS-CoV-2 with angiotensin-converting enzyme 2 (ACE-2) and transmembrane serine protease 2 (TMPRSS2) receptor has been implicated in other studies [8], and recent work by Rotondi et al. and Lazartigues et al. demonstrated the presence of TMPRSS2 mRNA and ACE-2 in thyroid cells [9,10]. Previously, in SARS-CoV and Middle East respiratory syndrome-related coronavirus (MERS-CoV), the same family, receptor-virus interaction has been described [11]. The abundance of these receptors in the thyroid [12] compared to other tissues (heart, adipose, and small intestine) potentially explains the subacute thyroiditis associated with SARS-CoV-2. This strengthens the mechanism of direct viral injury in SAT. Direct follicular damage presents the hyperthyroid state's clear clinical picture, such as anxiety, sweating, palpitations, and tachycardia. As documented above, our case also had tachycardia throughout the hospital stay (see Figure 4). Tachycardia could be related to initial hyperthyroidism or directly associated with COVID infection as the patient had also developed acute PE.

Medications used to treat patients with COVID-19, especially glucocorticoids and low molecular weight heparin, may also damage the gland and affect thyroid function [13]. Our patient also received dexamethasone and enoxaparin. Her thyroid dysfunction was detected before medication started; therefore, it is unlikely that drugs may have contributed to the gland damage. The direct effect of the SARS-CoV-2 virus is better implicated for the thyroid gland inflammation seen in this case.

In euthyroid patients with COVID-19 infection, studies report [14,15] a slight reduction in TSH and FT4 in patients with COVID-19 compared to non-COVID-19 cases. However, in most patients, SAT ensued after COVID-19 symptoms had disappeared, with a lag of 16 to 36 days. Neck pain radiating to the jaw and palpitations were the presenting symptoms in all patients [14]. Our patient did not present with pain, and the time profile is very different in that thyrotoxicosis presented at the same time as the acute phase of COVID infection. The thyrotoxicosis was transient, lasting only for about one week. These differentiate our patient from the typical painful and painless SAT reported. We think these are explainable by direct COVID-19 infection leading to transient and mild thyroid follicles destruction rather than post-infectious immune destruction.

A limitation of this case is that an USG of the thyroid gland was not done. The patient improved clinically while hospitalized and insisted on being discharged before USG thyroid could be done and was subsequently lost to follow-up.

## Conclusions

COVID-19-induced thyroiditis is an emerging phenomenon. Most cases resolve with supportive management. The exact mechanism of COVID-19-induced thyroiditis is still unknown. It is reassuring to see the resolution of thyroiditis within days of acute COVID-19 infection. Post-discharge clinical follow-ups of such patients would be beneficial in assessing the long-term effects of COVID-19 on the thyroid gland.

## References

[1] Desailloud R, Hober D (2009). Viruses and thyroiditis: an update. Virol J.

[2] Park SY, Kim EK, Kim MJ, Kim BM, Oh KK, Hong SW, Park CS (2006). Ultrasonographic characteristics of subacute granulomatous thyroiditis. Korean J Radiol.

[3] Intenzo CM, Park CH, Kim SM, Capuzzi DM, Cohen SN, Green P (1993). Clinical, laboratory, and scintigraphic manifestations of subacute and chronic thyroiditis. Clin Nucl Med.

[4] Scappaticcio L, Pitoia F, Esposito K, Piccardo A, Trimboli P (2021). Impact of COVID-19 on the thyroid gland: an update. Rev Endocr Metab Disord.

[5] Aemaz Ur Rehman M, Farooq H, Ali MM, Ebaad Ur Rehman M, Dar QA, Hussain A (2021). The association of subacute thyroiditis with COVID-19: a systematic review. SN Compr Clin Med.

[6] Croce L, Gangemi D, Ancona G, Liboà F, Bendotti G, Minelli L, Chiovato L (2021). The cytokine storm and thyroid hormone changes in COVID-19. J Endocrinol Invest.

[7] Wei L, Sun S, Xu CH (2007). Pathology of the thyroid in severe acute respiratory syndrome. Hum Pathol.

[8] Wang Q, Zhang Y, Wu L (2020). Structural and functional basis of SARS-CoV-2 entry by using human ACE2. Cell.

[9] Rotondi M, Coperchini F, Ricci G (2021). Detection of SARS-CoV-2 receptor ACE-2 mRNA in thyroid cells: a clue for COVID-19-related subacute thyroiditis. J Endocrinol Invest.

[10] Lazartigues E, Qadir MM, Mauvais-Jarvis F (2020). Endocrine significance of SARS-CoV-2's reliance on ACE2. Endocrinology.

[11] Li W, Moore MJ, Vasilieva N (2003). Angiotensin-converting enzyme 2 is a functional receptor for the SARS coronavirus. Nature.

[12] Li MY, Li L, Zhang Y, Wang XS (2020). Expression of the SARS-CoV-2 cell receptor gene ACE2 in a wide variety of human tissues. Infect Dis Poverty.

[13] Chen W, Tian Y, Li Z, Zhu J, Wei T, Lei J (2021). Potential interaction between SARS-CoV-2 and thyroid: a review. Endocrinology.

[14] Brancatella A, Ricci D, Viola N, Sgrò D, Santini F, Latrofa F (2020). Subacute thyroiditis after SARS-CoV-2 infection. J Clin Endocrinol Metab.

[15] Brancatella A, Ricci D, Cappellani D, Viola N, Sgrò D, Santini F, Latrofa F (2020). Is subacute thyroiditis an underestimated manifestation of SARS-CoV-2 infection? Insights from a case series. J Clin Endocrinol Metab.

